# Isolation and characterization of fourteen polymorphic microsatellite markers in the viperine snake *Natrix maura*

**DOI:** 10.1002/ece3.5623

**Published:** 2019-09-02

**Authors:** Hugo Le Chevalier, Neus Marí‐Mena, Belén Carro, Jérôme G. Prunier, Coralie Bossu, Elodie Darnet, Jérémie Souchet, Olivier Guillaume, Olivier Calvez, Romain Bertrand, Laurent Barthe, Gilles Pottier, Albert Martínez‐Sylvestre, Isabel Verdaguer‐Foz, Marc Mossoll‐Torres, Audrey Trochet, Fabien Aubret

**Affiliations:** ^1^ Station d'Ecologie Théorique et Expérimentale (SETE), UMR5321 Centre National de la Recherche Scientifique (CNRS) Moulis France; ^2^ Department of Molecular and Cell Biology, Evolutionary Biology Group (GIBE) Universidade da Coruña A Coruña Spain; ^3^ All Genetics & Biology SL A Coruña Spain; ^4^ Association Nature en Occitanie (NEO) Maison de l'Environnement de Midi‐Pyrénées Toulouse France; ^5^ Catalonia Reptile and Amphibian Rescue Center (CRARC) C/Santa Clara s/n Masquefa Spain; ^6^ Pirenalia Encamp Andorra; ^7^ Bomosa Les Escaldes Andorra

**Keywords:** *Natrix maura*, polymorphic microsatellites, population genetics, viperine snake

## Abstract

Nineteen polymorphic microsatellite loci were identified and developed for *Natrix maura*. Polymorphism was assessed for 120 individuals sampled across four sampling sites from the French Pyrenees Mountains. The number of alleles per locus ranged from 3 to 15, and expected heterozygosity per locus ranged from 0.227 to 0.863. We tested for deviation from Hardy–Weinberg equilibrium and linkage disequilibrium and assessed the presence of null alleles for all loci, resulting in a selection of 14 high‐quality polymorphic markers. These markers will be extremely useful in identifying fine‐scale genetic structures and providing insight into conservation management plans of this species.

## Graphical abstract

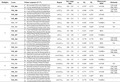

## 1 INTRODUCTION

Anthropogenic activities have already led to massive species extinction, and this loss of biodiversity is expected to continue at an unprecedented pace (Ceballos, Ehrlich, & Dirzo, 2017). Global warming is likely the most preoccupying threat given the potential synergy with many other environmental changes (Cahill et al., 2013; Thomas et al., 2004), impacting organisms at both the individual and population levels and resulting in local increase in extinction risks, species redistribution and community reshuffling (Aubret & Shine, 2010; Pauls, Nowak, Bálint, & Pfenninger, 2013; Walther et al., 2002). Ectotherms represent more than 98% of animal species and are the more likely to be affected because of direct physiological sensitivity to climate conditions (Deutsch et al., 2008; Dupoué et al., 2017; Sinervo et al., 2010). When the conservation status of a given population is uncertain, genetic studies constitute an indirect and valuable approach to assess the impacts of these environmental threats on levels of population genetic diversity and structure, effective dispersal, demographic status and possible past and future responses to global change (Segelbacher et al., 2010).

The viperine snake (*Natrix maura*) is a common Mediterranean snake inhabiting natural and artificial aquatic environments in Northwestern Africa, Iberian Peninsula, Southern France and Northern Italy. Although some localities may exhibit high snake densities, populations are generally considered as declining (Santos & Llorente, 2009). The viperine snake is threatened by multiple factors, such as aquatic pollution, habitat loss and fragmentation, direct destruction by humans because of confusion with venomous vipers and climate change (Santos & Fernández Cardenete, 2015; Santos & Llorente, 2009). All of these environmental threats are likely to interact significantly, impacting viperine snake populations (Gangloff, Sorlin, Cordero, Souchet, & Aubret, 2019; Muthoni, 2010). In this context, the development of polymorphic genetic markers is critical for this species in order to study patterns of genetic diversity and understand population structure and functioning. Here, we isolated and characterized 19 new polymorphic microsatellite markers for *N. maura* using Illumina high‐throughput sequencing.

## 2 MATERIAL & METHODS

We sampled DNA from 120 viperine snakes from four populations in the southwestern France (Ariège, Table 1), using buccal swabs as a noninvasive sampling method (Beebee, 2008). Swabs were suspended in 1X TE buffer for DNA conservation and DNA extraction was performed using the RealPure MicroSpin DNA Isolation Kit following manufacturers' instructions (Durviz). Microsatellite development was performed at AllGenetics (http://www.allgenetics.eu). A single DNA sample belonging to a female viperine snake was used to generate a library with the Nextera XT DNA Library Preparation Kit (Illumina). The library was then enriched in fragments with microsatellite motifs by hybridization to four groups of biotinylated oligo repeats (i.e., AC, AG, ACG, and ATCT) that were captured with Dynabeads/M280 Streptavidin (Invitrogen, Thermo Fisher Scientific). The enriched library was sequenced in the Illumina MiSeq PE300 platform (Macrogen Inc.). Reads were processed in Geneious 10.2.2 (Biomatters Ltd). Primer design was carried out in Primer3 software (Koressaar & Remm, 2007; Untergrasser et al., 2012) implemented in Geneious 10.2.2.

**Table 1:** Characteristics of sampled sites: name of the sampling site, geographic coordinates (WGS84), number of sampled individuals (*n*_ind_) per site

Sampling site	*X*	*Y*	*n*_ind_
Alas	42°57′00.208″N	1°02′46.365″E	32
Audressein	42°55′33.665″N	1°01′36.659″E	34
Augirein	42°55′53.390″N	0°54′58.164″E	22
Moulis	42°57′37.694″N	1°05′16.735″E	32

A total of 108 primer pairs, each targeting a different locus, were identified and organized into 31 multiplexes using Multiplex Manager (Holleley & Geerts, 2009). The computer‐designed multiplexes were validated and checked for polymorphism using DNA samples from an additional set of seven individuals. The polymerase chain reactions (PCRs) were carried out following Schuelke (2000). As oligonucleotide tails, we used the universal sequences M13 (GGA AAC AGC TAT GAC CAT), CAG (CAG TCG GGC GTC ATC), and T3 (AATTAA CCC TCA CTA AAGGG) labeled with the HEX dye, the FAM dye, and the TAMRA dye, respectively. PCRs were performed in a final reaction volume of 12.5 µl, containing around 10 ng of DNA, Type‐it Multiplex PCR Master Mix (Qiagen), and Primer Mix 1× (0.2 µM forward primers and labeled tails, and 0.02 µM reverse primers). The optimal PCR protocol consisted in an initial denaturation step at 95°C for 5 min, followed by 30 cycles of 95°C for 30 s, 57°C for 90 s, 72°C for 30 s; 8 cycles of 95°C for 30 s, 53°C for 90 s, 72°C for 30 s; and a final extension step at 68°C for 30 min. All PCR rounds included a negative control to check for potential cross‐contamination. PCR products were subsequently subjected to fragment analysis. Allele calling was performed using Geneious 11.1.2 (Biomatters). Finally, the 19 primer pairs with the highest polymorphism were organized into seven multiplexes according to dye colors and expected amplicon sizes (Table 2). We finally applied this genotyping protocol to the 120 viperine snake samples to assess markers' quality.

**Table 2:** Characteristics of the 19 microsatellites developed in the viperine snake (*Natrix maura*). The table provides multiplex and locus names, primer sequences, repeat motif and number, allelic size range (in base pairs), number of alleles (*n*_a_), observed and expected heterozygosity (*H*_O_ and *H*_E_, respectively), fluorescent label, and rationale for discarding (null alleles [NA] and linkage disequilibrium [LD]). The 14 high‐quality markers are indicated in bold

Multiplex	Locus	Primer sequence (5′−3′)	Repeat	Size range (bp)	*n*_a_	*H*_O_	*H*_E_	Fluorescent label	Rationale
1	**NM_064**	F: GCAAAGCTTCAACTGGCCAA	(AC)_12_	185–235	11	0.547	0.572	6‐FAM	LD (with NM_465)
		R: CCACAGGGTGACTATGGCTG							
	**NM_368**	F: CTGTGAAATGTTGGTGGCGC	(ATC)_15_	198–243	11	0.848	0.799	HEX	LD (with NM_321)
		R: CACATTGAAGTCCCGGGTGA							
2	**NM_268**	F: ACGGAAGTGACCCTCCAGTA	(AC)_14_	127–134	3	0.218	0.267	6‐FAM	–
		R: CGAAACGGTGGCACTGGATA							
	**NM_085**	F: GCTGGTTCCAGAAGGGTCTC	(AG)_15_	185–213	5	0.213	0.227	HEX	–
		R: TCCTTGGTGGGTCAAACTGG							
	NM_170	F: GCATCTTGAGCTCGTGAGGT	(AC)_30_	204–238	11	0.492	0.704	TAMRA	NA
		R: TCCGCCGATTCCAATTCCTT							
3	NM_384	F: GCCAAGGAACTGCTGAACCT	(AC)_17_	102–121	7	0.495	0.685	6‐FAM	NA
		R: CATTTGGGACTGGCAGCATG							
	**NM_462**	F: CACTAGTGGCAGCAGAGTGT	(AG)_12_	98–106	7	0.521	0.587	HEX	–
		R: TGGGCTGCAGAGATTCAGAG							
	**NM_497**	F: TTGCTTGCTGTGATGTGCTG	(AC)_17_	124–158	7	0.697	0.636	TAMRA	–
		R: ACGAAGTGTTGAGCGGAAGG							
	**NM_364**	F: AGAAGCAACCCAACACCAGA	(AC)_29_	191–214	13	0.778	0.778	HEX	LD (with NM_054)
		R: CTGCCATGGGTGTAGGACTG							
4	NM_013	F: GTCCTTTGGGAGAAGGGTGG	(AAAC)_15_	125–156	6	0.499	0.723	HEX	NA
		R: CCTTCTCCAGTGGTGGGTTC							
	**NM_214**	F: TATCTTTCCGGCTTTGCGGA	(AC)_24_	108–135	13	0.653	0.750	TAMRA	–
		R: TGCACAGTCACATGGAACCA							
	**NM_465**	F: TGCTTCTCTTGCCTCTTCGT	(AC)_17_	253–276	6	0.543	0.565	TAMRA	LD (with NM_064)
		R: AGCCACCACTCTGAGAGTCA							
5	NM_245	F: TGCGCCAAGAACAATCACAC	(AATAG)_11_	140–195	11	0.489	0.678	6‐FAM	NA
		R: TGCCACTCCACAACCAATCA							
	**NM_051**	F: CTTGCAACACAACGGAGTCG	(AC)_15_	126–132	3	0.486	0.528	TAMRA	–
		R: ACAACATCTGTGACGGCAGT							
6	NM_346	F: ATTGCTTGGCTTGGTTTGGC	(AAGG)_14_	190–292	15	0.445	0.863	6‐FAM	NA
		R: CCTAGAAATGAGGGCGGGAG							
	**NM_054**	F: GCCGCAAACCCAAACACTAG	(AC)_12_	138–229	11	0.519	0.627	HEX	LD (with NM_364)
		R: ACCAGTGATGGCGAACCTTT							
7	**NM_321**	F: TCGTGACAGTGAGTTGGCAG	(AAAG)_18_	129–183	12	0.771	0.774	6‐FAM	LD (with NM_368)
		R: TCTTTCCTCCTCTCCCTCCC							
	**NM_093**	F: CATGTGTCTGCCTGCATTGG	(AC)_7_	75–133	4	0.715	0.497	HEX	–
		R: CTTCATGTGGGATTGCGCTG							
	**NM_076**	F: ACCAGTTCACAAGTCCACGG	(ACCT)_18_	243–275	9	0.770	0.798	TAMRA	–
		R: AAAGAAGGATGCAGCGTGGA							

To avoid any bias in further analyses, we first identified populations showing HWE across the maximum number of markers. We used the *test_HW* function from the R‐distribution of the Genepop software (Rousset, 2008) to assess HWE for each locus and each population, and only retained loci showing HWE.

Considering each populations independently, we then estimated for each locus the number of alleles (*n*_a_), observed and expected heterozygosity (*H*_O_ and *H*_E,_ respectively) using FSTAT 2.9.3.2 (Goudet, 1995). We also computed null allele frequency along with 95% confidence intervals using the *null.all* function from the R‐package *PopGenReport* (Adamack & Gruber, 2014). Loci showing a lower bound exceeding a null allele frequency of 5% where discarded. We finally assessed linkage disequilibrium across populations using the *test_LD* function (Rousset, 2008). Tests for HWE and linkage disequilibrium were all conducted using false discovery rate FDR‐correction to account for multiple‐related tests (Benjamini & Hochberg, 1995).

## 3 RESULTS AND DISCUSSION

All loci amplified well (from 0% to 5.8% of missing values). Five loci (NM_013, NM_170, NM_346, NM_384, and NM_462) did not conform to HWE. Using a subset of the 14 remaining markers, we found that all populations conformed to HWE.

In each considered population, the 19 loci were found to be polymorphic with n_a_ ranging from 3 to 15 and H_E_ ranging from 0.227 to 0.863 (Table 2). Yet, the presence of null alleles was detected in five loci (NM_013, NM_170, NM_245, NM_346, and NM_384). These markers should therefore be used with caution as they can significantly affect the results of genetic analyses (Pompanon, Bonin, Bellemain, & Taberlet, 2005; Wen et al., 2013). They were discarded from further analyses, resulting in a new set of 14 polymorphic markers. Finally, we found significant linkage disequilibrium in three pairs of loci: NM_054/NM_364, NM_064/NM_465, and NM_321/NM_368. Some genetic analyses do not require linkage equilibrium (e.g., sPCA; Jombart, Devillard, Dufour, & Pontier, 2008), and we here provide a useful set of 14 polymorphic microsatellite markers for the viperine snake. For genetic analyses requiring independent loci, we recommend using makers showing highest levels of polymorphism (notably NM_465 in place of NM_064).

The viperine snake *N. maura* is a well‐suited model species as it is both common in Southwestern Europe while being threatened by multiple environmental factors, inducing distribution shifts and individual perturbations (Aubret & Shine, 2010; Muthoni, 2010). The new set of 14 high‐quality polymorphic markers developed in this study (Table 2, in bold) may be used in several scientific contexts from conservation surveys to population genetic studies.

## CONFLICT OF INTEREST

None declared.

## AUTHOR CONTRIBUTIONS

All authors contribute significantly to the present study and to the revision of the manuscript. H.L.C., with support from A.T., J.G.P., and F.A., wrote the manuscript. H.L.C. and E.D. performed DNA extractions and PCR. N.M.‐M. and B.C. performed sequencing, primer identification and selection. Statistical and genetic analyses were performed by J.G.P, A.T., H.L.C., and E.D. Animal captures and DNA sampling on the field were performed by H.L.C., E.D., C.B., J.S., O.G., O.C., R.B., L.B., G.P., A.M.‐S., I.V.‐F., M.M.‐T. Research project was leaded by F.A.

## Data Availability

The microsatellite data are available on Dryad: https://doi.org/10.5061/dryad.0vd1fj3
